# Impact of serum leptin and adiponectin levels on brain infarcts in patients with mild cognitive impairment and Alzheimer’s disease: a longitudinal analysis

**DOI:** 10.3389/fendo.2024.1389014

**Published:** 2024-04-15

**Authors:** Giovanni Carbone, Leonardo Bencivenga, Maria Angela Santoro, Natascia De Lucia, Maria Emiliana Palaia, Erica Ercolano, Francesco Scognamiglio, Paul Edison, Nicola Ferrara, Dino Franco Vitale, Giuseppe Rengo, Grazia Daniela Femminella

**Affiliations:** ^1^ Department of Translational Medical Sciences, “Federico II” University, Naples, Italy; ^2^ Department of Neurosciences, Reproductive and Odontostomatological Sciences, “Federico II” University, Naples, Italy; ^3^ Department of Brain Sciences, Imperial College London, London, United Kingdom; ^4^ Clinica San Michele, Maddaloni, CE, Italy; ^5^ Laboratorio di fisiopatologia del sistema neurovegetativo, Istituti Clinici Scientifici Maugeri Istituti di Ricovero e Cura a Carattere Scientifico (IRCCS) - Scientific Institute of Telese Terme, Telese Terme, BN, Italy

**Keywords:** leptin, adiponectin, brain infarcts, Alzheimer’s disease, MR imaging

## Abstract

**Introduction:**

The adipokines leptin and adiponectin have been associated with atherosclerosis and the risk of cerebral infarcts. Pre-clinical studies, however, suggest a protective role against ischemic brain damage. In this study we analyzed the relationship between serum leptin and adiponectin levels and the onset or progression of brain infarcts in subjects with mild cognitive impairment (MCI) and Alzheimer’s disease (AD).

**Methods:**

All data were extracted from the ADNI database. The final population included 566 subjects, with 58 healthy controls, 396 MCI and 112 AD. All patients with available serum leptin and adiponectin levels at baseline were selected. Demographics, neuropsychological test results, CSF biomarkers, regional brain metabolism with FDG-PET data and the number of brain infarcts on longitudinal MRI scans were extracted.

**Results:**

Leptin levels were significantly lower in patients with MCI than controls at baseline, while adiponectin levels were not different between the groups. Multivariate logistic regression analysis at baseline for the presence of brain infarcts showed a predictive value for leptin but not for adiponectin. Multivariate longitudinal analysis showed that age was the only significant predictor of brain infarcts development at 15-year follow-up, while serum leptin and adiponectin levels did not play a role in this population.

**Discussion:**

The evidence on the pathogenetic or protective role of adipokines on ischemic brain damage is mixed. In this MCI and AD population, serum leptin and adiponectin were not associated with the development of brain infarcts; therefore, these results do not support the use of adipokines as biomarkers of cerebrovascular pathology in this population.

## 1 Introduction

Alzheimer’s disease (AD) is the most common cause of neurodegenerative dementia and one of the most burdening diseases worldwide (1). The main pathophysiological hypothesis of AD is the amyloid-beta (Aβ) cascade (2), with abnormal phosphorylation of tau protein, resulting in the formation of neurofibrillary tangles, representing another hallmark of AD pathophysiology. Both Aβ-induced plaques formation and tau accumulation result in neuronal degeneration, and indeed these mechanisms constitute the bases of a recent biological classification of AD, relying on biomarkers evidence of amyloid, tau and neurodegeneration (3). However, other pathophysiological mechanisms are known to exert a role in AD development, with cerebrovascular disease (CVD) being a crucial one.

CVD comprises a series of different lesions, such as white matter hyperintensities, cerebral microbleeds, enlarged perivascular spaces and lacunar infarcts, visible on pathological examination and detectable *in vivo* with magnetic resonance imaging (MRI) (4). Vascular abnormalities have been reported in at least half of AD cases and have an additive effect on AD pathology (5). In particular, older subjects with silent brain infarcts on MRI have an increased risk of dementia and a steeper decline in cognitive function (6) and both large and small brain vessels diseases increase the likelihood of AD diagnosis (7). Clinicopathological studies have shown that silent brain infarcts lower the threshold for AD pathology to become clinically significant (8, 9). However, the mechanisms behind this increase in risk remain unclear (10).

Importantly, traditional cardiovascular risk factors such as obesity, hypertension, and diabetes can all increase the risk of cognitive decline and dementia (11). In particular, high midlife Body Mass Index (BMI), as well as central adiposity, results in a doubled risk of developing dementia in late life (12). The mechanisms underlying this increased risk might lie in the endocrine actions of the white adipose tissue, secreting hundreds of cell-signaling molecules, known as adipokines. Of those, leptin and adiponectin have been investigated as key mediators of the communication between periphery and the central nervous system (CNS) in healthy and pathological condition (13).

Leptin and adiponectin are adipocyte-derived hormones shown to have neuroprotective actions by modulating synaptic plasticity, improving learning and memory, and reducing Aβ levels and tau hyperphosphorylation in preclinical models (14, 15). Interestingly, leptin treatment has shown a neuroprotective effect in preclinical models of ischemic brain damage (16), while leptin deficiency resulted in larger infarct volume after focal cerebral ischemia in animal experiments (17). However, some epidemiological studies have found that high circulating leptin levels were associated with ischemic cerebrovascular disease (18, 19), while others have found no association (20). Low circulating adiponectin levels have been associated with ischemic cerebrovascular disease and with increased mortality after ischemic stroke (21, 22), although others have reported opposite findings (23).

Overall, evidence linking adipokines to CVD is controversial and it is not yet known what its actual contribution to AD pathophysiology could be. Here, we evaluated whether baseline serum leptin and adiponectin levels could predict the development of silent brain infarcts in subjects with MCI and AD from the Alzheimer’s Disease Neuroimaging Initiative (ADNI) cohort.

## 2 Methods

Data used in the preparation of this article were obtained from the Alzheimer’s Disease Neuroimaging Initiative (ADNI) database (adni.loni.usc.edu). The ADNI was launched in 2003 as a public-private partnership, led by Principal Investigator Michael W. Weiner, MD. The primary goal of ADNI has been to test whether serial magnetic resonance imaging (MRI), positron emission tomography (PET), other biological markers, and clinical and neuropsychological assessment can be combined to measure the progression of mild cognitive impairment (MCI) and early Alzheimer’s disease (AD). All ADNI studies are conducted according to the Good Clinical Practice guidelines, the Declaration of Helsinki, and U.S. 21 CFR Part 50 (Protection of Human Subjects), and Part 56 (Institutional Review Boards). Written informed consent was obtained from all participants. The ADNI protocol was approved by the Institutional Review Boards of all of the participating institutions. The ethics committees/institutional review board that approved the ADNI study are listed within **Supplementary Information File**.

### 2.1 Study population

The ADNI is a “longitudinal multicenter study designed to develop clinical, imaging, genetic, and biochemical biomarkers for the early detection and tracking of AD”(http://adni.loni.usc.edu/). ADNI enrols participants between the ages of 55 and 90: 1) Cognitively healthy Controls (HC) (MMSE 24-30, CDR of 0); 2) MCI subjects (MMSE 24-30, memory complaint, objective memory loss, CDR of 0.5, preserved activities of daily living); 3) Mild AD (MMSE 20-26, CDR of 0.5 or 1.0, NINCDS/ADRDA criteria for probable AD).

For this study we extracted data of controls, MCI and AD patients having been tested at baseline for serum biomarkers levels (leptin and adiponectin), brain MRI, neuropsychological tests (MMSE, NPI, ADAS-Cog, CDR and GDS), cerebrospinal fluid (CSF) tau, phosphorylated tau and amyloid. Brain 18F-FDG-PET was used to measure cerebral glucose metabolism and quantified using target-to-pons RATIO in the angular gyrus, bilateral posterior cingulate and temporal gyrus, as described. APOE4 positivity was defined as presence of one or more E4 allele.

### 2.2 Brain infarcts detection and serum adipokines assessment

The detection of cerebral infarcts in ADNI has been described by DeCarli et al. Briefly, adequately trained physicians performed brain infarct assessment over MRIs, magnifiable up to x3. Signal void, best seen in T2 weighted image was considered as indicator of vessel. Only images larger than 3mm were qualified as cerebral infarcts and, once detected, the location was marked in image space and transferred to the database as image coordinates, enabling for follow up review (24). MRI scans were repeated approximately every 12 months.

Serum samples collection for leptin and adiponectin dosage was performed at baseline visit, with the patient fasting and having fasted overnight. Serum leptin and adiponectin levels were determined by the ADNI Biomarkers Consortium using Luminex immunoassay technology, as part of a panel of 190 analytes with the Human Discovery Multi-Analyte Profile panel developed by Rules- Based Medicine (RBM, Austin, TX, USA) on the Luminex xMAP platform (25).

Further information about data collection can be found at http://adni.loni.usc.edu/wp-content/uploads/2010/09/ADNI_GeneralProceduresManual.pdf.

### 2.3 Statistical analysis

We used STATA 17 (StataCorp LLC) to analyze the data. Normality distribution was evaluated with Kolmogorov-Smirnov test. Continuous variables were expressed as mean ± SD. One-way ANOVA was used to compare the three groups (controls, MCI, AD) followed by a Bonferroni post-hoc correction. Categorical variables were compared by χ2 test. Multiple linear regression and multivariate logistic regression were performed to test associations between our variables of interest. The Cox proportional-hazards analysis was employed to identify the independent contribution of each factor associated with brain infarct development at follow up, based on a multivariate predictor model. For the multiple linear regression, logistic regression and Cox models, the functional form of the association between continuous factors and outcome was checked and modelled using a multivariable fractional polynomial (MFP) algorithm, as previously described (26). The MFP modelling algorithm (27), resulted in a final model that combines the exclusion of “unimportant” variables with the selection of a “reasonable” dose-response function for continuous variables. The *p*-value was set to ≤0.05 for variable selection and for testing between variable transformations. The relative weight of each significant factor in the final model was estimated by measuring the partial contribution to the global goodness-of-fit, as measured by the global R^2^ for the multiple regression model, by the McFadden’s global pseudo R^2^ for the logistic model and by the Royston & Sauerbrei’s R^2^_D_ for the Cox model (28). Their partition over the significant predictors was accomplished by the Shapley-Owen decomposition algorithm (29).

The internal validity of the models was computed by assessing the stability of each model characteristics using non-parametric bootstrap sampling. The stability of each factor tested in the model was measured by the frequency that this factor was selected as “significant” in a large series (1000) of bootstrap replications of the dataset. Each bootstrapped dataset may be considered as a random replicate of the original dataset; thus, the bootstrap inclusion frequency (BIF) of a given factor in the final model represents the confidence we can place on its association with the outcome (stability), considering the expected random variability of the data (30).

## 3 Results

The cohort of subjects extracted from ADNI database for this study consisted of 566 individuals, of which 58 HC, 396 patients diagnosed with MCI and 112 AD patients. The demographic features and baseline biomarkers levels of the entire population are summarized in **Table 1**. Overall, the study population mean age was 74.8 ± 7.4 years and comprised 38% of female. The three groups were homogenous in terms of age, gender, education levels and BMI. The proportion of ApoE4 carriers was significantly higher in AD group compared to both HC and MCI, as well as in MCI compared to HC, as expected. The prevalence of the most common cardiovascular risk factors (smoke, diabetes, hypertension, dyslipidaemia, atrial fibrillation) was not different among the three groups.

**Table 1:** Baseline characteristics of subjects.

Variables	ALL (n=566)	HC (n=58)	MCI (n=396)	AD (n=112)
**Age, years**	74,8 ± 7,4	75,1± 5,8	74,8± 7,4	74,9 ± 8
**Women/Men (% of dx)**	215 (38)/351 (62)	28 (48,3)/30 (51,7)	140 (35,4)/256 (64,6)	47 (42)/65 (58)
**BMI, Kg/m²**	26 ± 4	27 ± 4	26 ± 4	26 ± 4
**Education level, years**	15,5 ± 3	15,6 ± 2,7	15,6 ± 3	15,1 ± 3,2
**Smoker yes/no (% of dx)**	237 (41,9)/329 (58,1)	29 (50)/29 (50)	163 (41,2)/233 (58,8)	45 (40,2)/67 (59,8)
**Dyslipidemia yes/no (% of dx)**	281 (49,6)/285 (50,4)	30 (51,7)/28 (48,3)	192 (48,5)/204 (51,5)	59 (52,7)/53 (47,3)
**Hypertension yes/no (% of dx)**	308 (54,4)/258 (45,6)	34 (58,6)/24 (41,4)	214 (54)/182 (46)	60 (53,6)/52 (46,4)
**Diabetes yes/no (% of dx)**	49 (8,7)/517 (91,3)	5 (8,3)/53 (91,4)	38 (9,6)/358 (90,4)	6 (5,4)/106 (94,6)
**Atrial fibrillation yes/no (% of dx)**	24 (4,2)/542 (95,8)	1 (1,7)/57 (98,3)	20 (5,1)/376 (94,9)	3 (2,7)/109 (97,3)
**ApoE4 carrier (% of dx)**	292 (51,6)	5(8,6)	211 (53,3) *	76 (67,9) *#
**MMSE**	26,5 ± 2,4	28,9 ± 1,2	27,0 ± 1,8 *	23,6 ± 1,9 *#
**NPI**	1,9 ± 2,8	0,3 ± 0,7	1,9 ± 2,7 *	3,4 ± 3,3 *#
**ADAS COG**	12,3 ± 5,8	6,3 ± 2,8	11,5 ± 4,4 *	18,3 ± 6,4 *#
**CDR**	0,5 ± 0,2	0 ± 0	0,5 ± 0,0 *	0,7 ± 0,3 *#
**GDS**	1,5 ± 1,4	0,9 ± 1,2	1,6 ± 1,4 *	1,7 ± 1,4 *
**Amyloid Beta CSF** **pg/ml**	930,7 ± 583,3	1653,9 ± 592,9	852,6± 487,2 *	655,3± 374,3 *#
**TAU CSF** **pg/ml**	308,7 ± 125,8	225,7 ± 73,11	308,9 ± 123,3 *	357,5 ± 130,4 *#
**Phospho-TAU CSF** **pg/ml**	30,4 ± 14,6	19,9 ± 6,5	30,6 ± 14,4 *	36,2 ± 15,3 *#
**FDG Temporal Left SUVR**	1,14 ± 0,15	1,25 ± 0,12	1,16 ± 0,14 *	1,04 ± 0,16 *#
**FDG Temporal Right SUVR**	1,14 ± 0,14	1,22 ± 0,11	1,15 ± 0,12	1,05 ± 0,15 *#
**FDG Angular Left SUVR**	1,18 ± 0,17	1,32 ± 0,14	1,19 ± 0,15 *	1,08 ± 0,17 *#
**FDG Angular Right SUVR**	1,19 ± 0,17	1,32 ± 0,17	1,20 ± 0,15 *	1,08 ± 0,16 *#
**FDG Cingulum Post Bilateral SUVR**	1,27 ± 0,18	1,40 ± 0,15	1,28 ± 0,17 *	1,16 ± 0,14 *#
**Cerebral Infarcts yes/no (% of dx)**	42 (7,4)/524 (92,6)	4 (6,9)/54 (93,1)	31 (7,8)/365 (92,2)	7 (6,3)/105 (93,8)
**Serum leptin (ng/ml)**	0.94 ± 0.42	1.10 ± 0.42	0.92 ± 0.41*	0.96 ± 0.44
**Serum adiponectin (μg/ml)**	0.76 ± 0.25	0.70 ± 0.29	0.76 ± 0.25	0.80 ± 0.25

*Significantly different vs. CTR (p<.05); # Significantly different vs. MCI (p<.05).

Data are expressed as mean ± SD.

As expected, the neuropsychometric tests showed worse cognitive performance and behavioral symptoms in AD compared to MCI and HC, and in MCI compared to HC.

As for the CSF biomarkers, levels of Aβ were significantly lower in in AD compared to both HC and MCI and in MCI compared to HC. An opposite trend (higher levels) was observed for tau and phospho-tau. Brain metabolism evaluated by FDG-PET in the main regions of interest (temporal lobes and angular right and left gyri and at the posterior bilateral cingulum), was significantly reduced in AD and MCI compared to HC, except for the temporal right lobe which did not show significantly different uptake in MCI vs HC.

On average, 7.4% of the whole population had at least one detectable brain infarct on MRI. The mean number of infarcts was not significantly different among the three groups.

We evaluated whether the levels of serum adipokines leptin and adiponectin differed among the three groups. Only serum leptin levels were significantly lower in MCI compared to HC. Adiponectin levels were similar among the three groups (**Table 1**). In the whole study population, an inverse correlation was observed between adiponectin and leptin levels (Pearson’s r = −0.18 [− 0.25–0.09] p < 0.00).

Since it has been demonstrated that leptin and adiponectin levels tend to be generally higher in women than in men (31), we wanted to test whether this could be the case also in our study population. Therefore, we stratified our three groups by gender and compared serum leptin and adiponectin levels between men and women within each diagnostic group. As expected, both adipokines levels were significantly higher in women in each of the three groups, as shown in **Supplementary Figure 1.**

To establish which are the variables that could be associated with baseline serum leptin levels in our population, we performed a multivariate regression analysis for leptin levels in our disease group (MCI and AD), building a model that included age, gender, diagnosis, BMI, ADAS-Cog, MMSE, dyslipidaemia, hypertension and brain infarcts. The variables that showed a significant association with serum leptin levels in our population were age, gender, BMI, MMSE and hypertension as shown in **Supplementary Table 1**. A good fraction of the variability in plasma leptin levels was explained by this model, as indicated by the global *R^2^* of 0.58. A large fraction of this contribution is shared by both gender and BMI as indicated by the relative contribution to the global *R^2^* (45.5% and 48.5% respectively), while a marginal impact on the association is evidenced by the other 3 significant factors (age, MMSE and hypertension) as shown by their almost irrelevant, albeit statistically significant, contribution to *R^2^*.

Similarly, we performed a multivariate regression analysis with the same predictors for adiponectin levels, showing that age, gender, BMI and dyslipidaemia were associated with baseline adiponectin levels with a global *R^2^* of 0.16. As for leptin, a large fraction of the *R^2^* is related to the gender and BMI partial contribution (**Supplementary Table 2**).

To evaluate the influence of leptin and adiponectin on the presence of brain infarcts in AD and MCI subjects, we built two multivariable fractional polynomial logistic regression models for the presence or absence of silent brain infarct at baseline. The model for leptin included as independent predictors age, gender, leptin levels, diagnosis, BMI, diabetes, dyslipidaemia, hypertension, smoke and atrial fibrillation. The model proved significant, with and overall modest pR^2 =^ 0.07, with age and leptin being the only significant predictors of brain infarcts at baseline, as shown in **Table 2**. Of note, as indicated by the percent of contribution to the global pR2, the greatest fraction is attributable to age (81.6%), leaving to leptin a modest contribution (18.4%).

**Table 2:** Logistic regression of leptin versus baseline infarcts in MCI and AD subjects.

	B	Odds= Exp (B)	pR2c (%)	95%CI	BIF (%)
**Age (10 years)**	.924*	**2.52**	**81.6**	**1.480, 4.306**	**92.8**
Gender	.277	1.34		.525, 3.416	5.1
**Leptin**	.963*	**2.62**	**18.4**	**1.140, 6.002**	**63.9**
Definitive diagnosis	-.400	0.67		.277, 1.620	4.6
BMI	.058	1.06		.955, 1.188	7.5
Diabetes	-1.609	0.20		.264, 1.595	4.0
Dyslipidemia	.270	1.31		.651, 2.635	4.3
Hypertension	.029	1.03		.499, 2.150	1.7
Smoke	-.274	0.76		.363, 1.530	3.0
Atrial fibrillation	-.579	0.56		.121, 2.612	0.4

pR^2^ = 0.07 (Cox & Snell); Model χ2 (2) = 18.27, p <.01.

* p<.05.

pR^2^c = global pseudo R2 contribution; BIF = Bootstrap Inclusion Frequency based on 1000 bootstrap sample.

Significant variables (p<.05) are in bold.

Similarly to leptin we built a logistic model for the presence of baseline brain infarcts, using age, gender, adiponectin levels, diagnosis, BMI, diabetes, dyslipidaemia, hypertension, smoke and atrial fibrillation as independent predictors. The overall model proved significant (χ2 (1) =12.96, p<.01), with age (odds ratio of 2.45), being the only significant predictor of brain infarcts at baseline (**Table 3**).

**Table 3:** Logistic regression of adiponectin versus baseline infarcts in MCI and AD subjects.

	B	Odds= Exp (B)	pR2c (%)	95%CI	BIF (%)
**Age (10 years)**	.896*	**2.45**	**100**	**1.450, 4.140**	**93.4**
Gender	.482	1.62		.809, 3.383	20.8
Adiponectin	.476	1.61		.358, 7.945	3.0
Definitive diagnosis	-.385	0.68		.279, 1.630	4.2
BMI	.113	1.10		1.012, 1.206	26.0
Diabetes	-1.61	0.20		.025, 1.561	5.8
Dyslipidemia	.322	1.38		.678, 2.803	4.7
Hypertension	.076	1.08		.526, 2.227	2.1
Smoke	-.287	0.75		.367, 1.538	5.2
Atrial fibrillation	-.597	0.55		3.22e-06,.038	0.6

R^2^ = 0.05 (Cox & Snell); Model χ2 (1) = 12.96, p <.01.

* p<.05.

pR^2^c = global pseudo R2 contribution; BIF = Bootstrap Inclusion Frequency based on 1000 bootstrap sample.

Significant variables (p<.05) are in bold.

**Figure 1:**
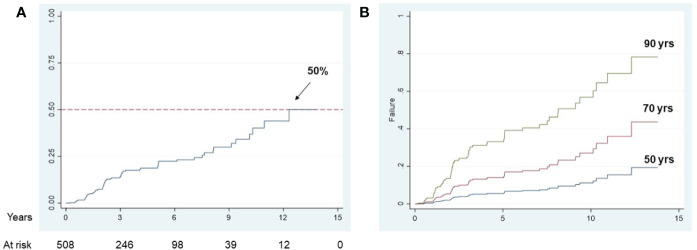
Longitudinal brain infarct incidence in the study population Kaplan-Meier curve for overall brain infarct incidence in MCI and AD subjects **(A)**; population adjusted curves, estimated at specific age values, showing the brain infarcts incidence that would be observed if all subjects in the study population had the given specific factor value **(B)**.

Finally, to establish whether circulating adipokines might influence the development of brain infarcts in this cohort of 508 MCI and AD subjects, we conducted a multivariable fractional polynomial Cox regression analysis with age, gender, baseline leptin and adiponectin levels, BMI, hypertension and ApoE4 carrier status as predictors (**Table 4**). 83 new brain infarcts were observed for a period of 15 years. The Cox analysis showed that only age was a significant predictor for the development of brain infarcts in this population (hazard ratio 1.63). At a maximum follow up time of 15 years, 50% of the population had developed at least one brain infarct (**Figure 1A**), and the effect of age on brain infarct incidence at follow up is shown if **Figure 1B**. After adjusting for all the variables included in the Cox regression model, an increase in age by 10 years would increase the risk of developing a brain infarct by 63%. In the representative **Figure 1B**, while the risk of developing a brain infarct at 50 years of age is 20%, at 90 years of age it almost reaches 80% in this study population.

**Table 4:** Multivariate Cox regression analysis on the development of brain infarcts.

	Hazard ratio	p-value	R^2^_D_c (%)	95%CI	BIF (%)
**Age (10 years)**	**1.63**	**0.005**	**100**	**1.16-2.28**	**85.9**
Gender	0.94	0.848	–	0.51-1.72	3.0
Leptin	1.24	0.592	–	0.56-2.73	17.6
Adiponectin	1.48	0.434	–	0.55-4.02	10.3
BMI	1.01	0.835	–	0.94-1.08	4.8
Hypertension	1.54	0.068	–	0.97-2.47	30.2
ApoE4 carrier	0.96	0.882	–	0.62-1.51	2.0

global R^2^_D_=0.13.

R^2^_D_c =global R^2^_D_c contribution; BIF = Bootstrap Inclusion Frequency based on 1000 bootstrap sample.

Significant variables (p<.05) are in bold.

## 4 Discussion

The focus of this work has been on the role of two adipokines, leptin and adiponectin, as potential CVD biomarkers in the AD continuum. We showed that leptin levels at baseline were associated with the presence of brain infarcts and, although we failed to demonstrate a predictive value of serum leptin and adiponectin on brain infarcts development in this population, our findings have shed further light on this topic, which is still much debated.

Leptin is implicated in body weight homeostasis by regulating feeding behavior, energy expenditure, thermogenesis, autonomic function, and systemic metabolism (13). Leptin has been shown to have a neuroprotective effect in experimental models (32). In clinical AD studies though, evidence is controversial, as some studies have shown decreased leptin levels in CSF and plasma in AD patients, while others have shown increased or unaffected levels (33–35). Leptin treatment ameliorates ischemic damage in models of oxygen-glucose deprivation *in vitro* and in animal models of cerebral ischemia (36). However, several epidemiological studies have found that high circulating leptin levels are associated with ischemic cerebrovascular disease (20).

Adiponectin modulates inflammatory responses, energy expenditure, central food intake, and several metabolic processes (12). Low circulating adiponectin levels have been associated with heart failure, coronary artery disease, dyslipidemia, and type 2 diabetes (11). Low circulating levels of this adipokine have been associated with ischemic cerebrovascular disease (37), and with increased mortality after ischemic stroke, predicting neurological sequelae severity and functional outcome (23). Adiponectin has important anti-inflammatory and anti-atherogenic effects by suppressing the production of pro-inflammatory cytokines and modulating the expression of anti-inflammatory cytokines on different cell types. Moreover, adiponectin is considered as an immune mediator because it is significantly involved in innate and adaptative immune response (38).

In our population, adiponectin levels did not significantly differ among the three diagnostic groups. However, leptin levels were lower in MCI compared to controls. This is consistent with previous findings reported by Holden et al. who suggested higher leptin concentration were correlated with a less expressed cognitive decline in the elderly in a prospective, longitudinal cohort study (39). The data from Khemka also confirmed that lower levels of leptin were found in subjects with both AD and MCI compared with controls (33). Our data suggest a trend towards higher levels of adiponectin with increasing cognitive decline, however larger populations studies might highlight any significant differences. Some studies have shown a significant positive association between higher serum adiponectin levels and better cognitive function in postmenopausal women (40), while other reports suggest that in AD patients increased adiponectin serum levels could suggest a compensatory mechanism against neurodegeneration (38).

Consistent with previous findings, leptin and adiponectin levels in our study population were significantly higher in women than men (31). Indeed, the sexual dimorphism of leptin concentration is not uncommon in animal and human studies. It has been shown that circulating leptin concentration was significantly higher in women with heart failure or diabetes than men (41). Although the detailed mechanism is not yet fully understood, the significant difference in leptin concentrations in diabetic women might be explained by a higher proportion of body fat in women and the counter-regulatory effect between leptin and testosterone (42), high level of adiponectin in female, as well as high oestrogen that functions as negative regulator of leptin (43).

Serum leptin levels in our study population were significantly associated with gender, BMI and hypertension, in accordance with previous evidence on other populations (44, 45). Similarly, baseline adiponectin levels were associated with age, gender, BMI and dyslipidaemia.

In our study, we focused on the association of circulating adipokines with cerebrovascular pathology in MCI and AD. As Alzheimer’s clinical syndrome is multifactorial and might be due to mixed pathologies (46), other factors than amyloid and tau will likely contribute to and/or modify onset and progression of symptoms. Among these factors, CVD plays a critical role (47). Neuropathological studies have shown that cerebrovascular pathology is a major risk factor for clinically diagnosed AD-type dementia (48). A large autopsy-based neuropathological study importantly revealed that 80% of patients diagnosed with AD and no evidence of mixed (vascular) dementia had vascular pathology including cortical infarcts, lacunes, cerebral microbleeds, and multiple microinfarcts indicative of small vessel disease (SVD), intracranial atherosclerosis, arteriolosclerosis, perivascular spacing, and cerebral amyloid angiopathy, supporting the concept that cerebrovascular dysfunction is prominent in AD and lowers the threshold for dementia for a given AD pathology burden (49). In our population, 7.4% of participants had at least one brain infarct detected on brain MRI. At baseline, leptin levels were significantly associated with the presence of brain infarcts. The prevalence of brain infarcts in the ADNI population is generally lower compared to other cohorts; this might limit the power to find significant associations with adipokine levels and it might be because the prevalence of cardiovascular risk factors is generally lower in the ADNI cohort compared to the American population. However, our analysis did not find significant association between cardiovascular risk factors and brain infarcts, both at baseline and at the longitudinal analysis since age was the only predictor of brain infarcts in this population.

This work has some limitations: body fat and other parameters (e.g., insulin, testosterone, oestrogen levels), which may also play an important role on leptin, adiponectin and cognitive decline, were not collected. Moreover, leptin and adiponectin levels were only measured at baseline. We evaluated serum leptin and adiponectin levels; however, these might not reflect central adipokines levels, therefore CSF leptin and adiponectin should also be evaluated. Longitudinal cohort studies with larger sample size, different ages, and different vascular risk factors are required to comprehensively expand the understanding of the role of adipokines in AD. A strength of this work is represented by the fact that the ADNI cohort is well characterized in terms of biomarkers, and with a long follow up time.

### 4.1 Conclusions and implications

In a population of MCI, AD and healthy control patients, we sought to analyze the relationship that serum adipokines levels have with silent cerebral ischemic events. This is relevant since the correlation between adipokines levels and the progression of the AD due to its cerebrovascular component is still controversial.

The results of the present research suggest that serum leptin is associated with the presence of brain infarcts at baseline in patients with MCI and AD, however neither leptin nor adiponectin are associated with the development of cerebral infarcts. Although this might be limited by the small statistical power due to the low number of cerebral infarcts in our study population, we must conclude that at present leptin and adiponectin cannot be considered predictive markers of cerebrovascular disease in this population.

Extending the analysis to larger populations with cardiovascular risk factors prevalence which could be more like the general population, might help better clarify the role of adipokines in the pathogenesis of cerebrovascular disease in the AD continuum.

## Author’s note

Data used in preparation of this article were obtained from the Alzheimer’s Disease Neuroimaging Initiative (ADNI) database (http://adni.loni.usc.edu). As such, the investigators within the ADNI contributed to the design and implementation of ADNI and/or provided data but did not participate in analysis or writing of this report. A complete listing of ADNI investigators can be found at: http://adni.loni.usc.edu/wp-content/uploads/how to apply/ADNI Acknowledgement List.pdf.

## Data availability statement

Publicly available datasets were analyzed in this study. This data can be found here: http://adni.loni.usc.edu.

## Ethics statement

The studies involving humans were approved by Alzheimer’s Disease Neuroimaging Initiative (ADNI) Ethics approval. The studies were conducted in accordance with the local legislation and institutional requirements. The participants provided their written informed consent to participate in this study.

## Author contributions

GC: Writing – original draft. LB: Writing – original draft. MS: Writing – original draft. ND: Writing – review & editing. MP: Writing – review & editing. EE: Writing – review & editing. FS: Writing – review & editing. PE: Writing – review & editing. NF: Writing – review & editing. DV: Writing – review & editing. GR: Writing – review & editing. GF: Conceptualization, Writing – review & editing.

## References

[1] Scheltens P De Strooper B Kivipelto M Holstege H Chetelat G Teunissen CE. Alzheimer’s disease. Lancet. (2021) 397:1577–90. doi: 10.1016/S0140-6736(20)32205-4 PMC8354300 33667416

[2] Tolar M Abushakra S Sabbagh M. The path forward in Alzheimer’s disease therapeutics: Reevaluating the amyloid cascade hypothesis. Alzheimers Dement. (2020) 16:1553–60. doi: 10.1016/j.jalz.2019.09.075 31706733

[3] Jack CR Jr. Bennett DA Blennow K Carrillo MC Dunn B Haeberlein SB. NIA-AA Research Framework: Toward a biological definition of Alzheimer’s disease. Alzheimers Dement. (2018) 14:535–62. doi: 10.1016/j.jalz.2018.02.018 PMC5958625 29653606

[4] Kim HW Hong J Jeon JC. Cerebral small vessel disease and Alzheimer’s disease: A review. Front Neurol. (2020) 11:927. doi: 10.3389/fneur.2020.00927 32982937 PMC7477392

[5] Pantoni L. Not-so-silent infarcts. Lancet Neurol. (2003) 2:335. doi: 10.1016/S1474-4422(03)00406-X 12849148

[6] Vermeer SE Prins ND den Heijer T Hofman A Koudstaal PJ Breteler MM. Silent brain infarcts and the risk of dementia and cognitive decline. N Engl J Med. (2003) 348:1215–22. doi: 10.1056/NEJMoa022066 12660385

[7] Arvanitakis Z Capuano AW Leurgans SE Bennett DA Schneider JA. Relation of cerebral vessel disease to Alzheimer’s disease dementia and cognitive function in elderly people: a cross-sectional study. Lancet Neurol. (2016) 15:934–43. doi: 10.1016/S1474-4422(16)30029-1 PMC4969105 27312738

[8] Roman GC. Brain infarction and the clinical expression of Alzheimer disease. JAMA. (1997) 278:113–4. doi: 10.1001/jama.1997.03550020045023 9214520

[9] Jellinger KA Mitter-Ferstl E. The impact of cerebrovascular lesions in Alzheimer disease–a comparative autopsy study. J Neurol. (2003) 250:1050–5. doi: 10.1007/s00415-003-0142-0 14504965

[10] Vermeer SE Longstreth WT Jr. Koudstaal PJ. Silent brain infarcts: a systematic review. Lancet Neurol. (2007) 6:611–9. doi: 10.1016/S1474-4422(07)70170-9 17582361

[11] Ishii M Iadecola C. Adipocyte-derived factors in age-related dementia and their contribution to vascular and Alzheimer pathology. Biochim Biophys Acta. (2016) 1862:966–74. doi: 10.1016/j.bbadis.2015.10.029 PMC4821723 26546479

[12] Kiliaan AJ Arnoldussen IA Gustafson DR. Adipokines: a link between obesity and dementia? Lancet Neurol. (2014) 13:913–23. doi: 10.1016/S1474-4422(14)70085-7 PMC4228955 25142458

[13] Forny-Germano L De Felice FG Vieira M. The role of leptin and adiponectin in obesity-associated cognitive decline and Alzheimer’s disease. Front Neurosci. (2018) 12:1027. doi: 10.3389/fnins.2018.01027 30692905 PMC6340072

[14] Shanley LJ Irving AJ Harvey J. Leptin enhances NMDA receptor function and modulates hippocampal synaptic plasticity. J Neurosci. (2001) 21:RC186. doi: 10.1523/JNEUROSCI.21-24-j0001.2001 11734601 PMC6763052

[15] Perez-Gonzalez R Alvira-Botero MX Robayo O Antequera D Garzon M Martin-Moreno AM. Leptin gene therapy attenuates neuronal damages evoked by amyloid-beta and rescues memory deficits in APP/PS1 mice. Gene Ther. (2014) 21:298–308. doi: 10.1038/gt.2013.85 24430238

[16] Zhang J Deng Z Liao J Song C Liang C Xue H. Leptin attenuates cerebral ischemia injury through the promotion of energy metabolism via the PI3K/Akt pathway. J Cereb Blood Flow Metab. (2013) 33:567–74. doi: 10.1038/jcbfm.2012.202 PMC3618393 23299243

[17] Terao S Yilmaz G Stokes KY Ishikawa M Kawase T Granger DN. Inflammatory and injury responses to ischemic stroke in obese mice. Stroke. (2008) 39:943–50. doi: 10.1161/STROKEAHA.107.494542 18239178

[18] Soderberg S Stegmayr B Ahlbeck-Glader C Slunga-Birgander L Ahren B Olsson T. High leptin levels are associated with stroke. Cerebrovasc Dis. (2003) 15:63–9. doi: 10.1159/000067128 12499713

[19] Menon B Krishnan R. Role of leptin in acute ischemic stroke. J Neurosci Rural Pract. (2018) 9:376–80. doi: 10.4103/jnrp.jnrp_5_18 PMC6050775 30069095

[20] Saber H Himali JJ Shoamanesh A Beiser A Pikula A Harris TB. Serum leptin levels and the risk of stroke: the framingham study. Stroke. (2015) 46:2881–5. doi: 10.1161/STROKEAHA.115.009463 PMC4589501 26337973

[21] Yang Y Hu W Jiang S Wang B Li Y Fan C. The emerging role of adiponectin in cerebrovascular and neurodegenerative diseases. Biochim Biophys Acta. (2015) 1852:1887–94. doi: 10.1016/j.bbadis.2015.06.019 26118345

[22] Sasaki M Kawano T Saito T Yuzawa M Saito T Ikoma A. Hypoadiponectinemia in patients with cerebral infarction: comparison with other atherosclerotic disorders. Am J Med Sci. (2007) 333:140–4. doi: 10.1097/MAJ.0b013e318031b7af 17496731

[23] Wang Z Li B Wang Y Maimaitili A Qin H Dangmurenjiafu G. The association between serum adiponectin and 3-month outcome after ischemic stroke. Cardiovasc Diabetol. (2019) 18:105. doi: 10.1186/s12933-019-0908-z 31412946 PMC6694580

[24] DeCarli C Carmichael O He J. MRI infarct assessment in ADNI (2013). Available online at: http://adniloniuscedu/.

[25] Consortium’ TB. Biomarkers Consortium Plasma Proteomics Project RBM multiplex data (2013). Available online at: http://adniloniuscedu/.

[26] Lanzillo B Piscosquito G Marcuccio L Lanzillo A Vitale DF. Prognosis of severe acquired brain injury: Short and long-term outcome determinants and their potential clinical relevance after rehabilitation. A comprehensive approach to analyze cohort studies. PloS One. (2019) 14:e0216507. doi: 10.1371/journal.pone.0216507 31557186 PMC6762165

[27] Royston P Ambler G Sauerbrei W. The use of fractional polynomials to model continuous risk variables in epidemiology. Int J Epidemiol. (1999) 28:964–74. doi: 10.1093/ije/28.5.964 10597998

[28] Royston P Sauerbrei W. A new measure of prognostic separation in survival data. Stat Med. (2004) 23:723–48. doi: 10.1002/sim.1621 14981672

[29] Shorrocks AF. Decomposition procedures for distributional analysis: a unified framework based on the Shapley value. J Econ Inequal. (2013) 11:99–126. doi: 10.1007/s10888-011-9214-z

[30] Royston P Sauerbrei W. Multivariate model building. A pragmatic approach to regression analysis based on fractional polynomials for modeling continuous variables. Chichester, UK: Wiley (2008).

[31] Jequier E. Leptin signaling, adiposity, and energy balance. Ann N Y Acad Sci. (2002) 967:379–88. doi: 10.1111/j.1749-6632.2002.tb04293.x 12079865

[32] McGregor G Harvey J. Food for thought: Leptin regulation of hippocampal function and its role in Alzheimer’s disease. Neuropharmacol. (2018) 136:298–306. doi: 10.1016/j.neuropharm.2017.09.038 28987937

[33] Khemka VK Bagchi D Bandyopadhyay K Bir A Chattopadhyay M Biswas A. Altered serum levels of adipokines and insulin in probable Alzheimer’s disease. J Alzheimers Dis. (2014) 41:525–33. doi: 10.3233/JAD-140006 24625795

[34] Johnston JM Hu WT Fardo DW Greco SJ Perry G Montine TJ. Low plasma leptin in cognitively impaired ADNI subjects: gender differences and diagnostic and therapeutic potential. Curr Alzheimer Res. (2014) 11:165–74. doi: 10.2174/1567205010666131212114156 PMC4040126 24359504

[35] Yin H Tian S Huang R Cai R Guo D Lin H. Low plasma leptin and high soluble leptin receptor levels are associated with mild cognitive impairment in type 2 diabetic patients. Front Aging Neurosci. (2018) 10:132. doi: 10.3389/fnagi.2018.00132 29867443 PMC5962657

[36] Zhang F Wang S Signore AP Chen J. Neuroprotective effects of leptin against ischemic injury induced by oxygen-glucose deprivation and transient cerebral ischemia. Stroke. (2007) 38:2329–36. doi: 10.1161/STROKEAHA.107.482786 17600230

[37] Katsiki N Mantzoros C Mikhailidis DP. Adiponectin, lipids and atherosclerosis. Curr Opin Lipidol. (2017) 28:347–54. doi: 10.1097/MOL.0000000000000431 28463859

[38] Polito R Di Meo I Barbieri M Daniele A Paolisso G Rizzo MR. Adiponectin role in neurodegenerative diseases: focus on nutrition review. Int J Mol Sci. (2020) 21, 9255. doi: 10.3390/ijms21239255 33291597 PMC7729837

[39] Holden KF Lindquist K Tylavsky FA Rosano C Harris TB Yaffe K. Serum leptin level and cognition in the elderly: Findings from the Health ABC Study. Neurobiol Aging. (2009) 30:1483–9. doi: 10.1016/j.neurobiolaging.2007.11.024 PMC5278645 18358569

[40] De Franciscis P Barbieri M Leo S Dalise AM Sardu C Marfella R. Serum adiponectin levels are associated with worse cognitive function in postmenopausal women. PloS One. (2017) 12:e0186205. doi: 10.1371/journal.pone.0186205 29267309 PMC5739380

[41] Park HK Ahima RS. Physiology of leptin: energy homeostasis, neuroendocrine function and metabolism. Metabolism. (2015) 64:24–34. doi: 10.1016/j.metabol.2014.08.004 25199978 PMC4267898

[42] Cammisotto PG Bukowiecki LJ. Mechanisms of leptin secretion from white adipocytes. Am J Physiol Cell Physiol. (2002) 283:C244–50. doi: 10.1152/ajpcell.00033.2002 12055093

[43] Xing Y Liu J Xu J Yin L Wang L Li J. Association between plasma leptin and estrogen in female patients of amnestic mild cognitive impairment. Dis Markers. (2015) 2015:450237. doi: 10.1155/2015/450237 26693203 PMC4677007

[44] Sawaguchi T Nakajima T Haruyama A Hasegawa T Shibasaki I Nakajima T. Association of serum leptin and adiponectin concentrations with echocardiographic parameters and pathophysiological states in patients with cardiovascular disease receiving cardiovascular surgery. PloS One. (2019) 14:e0225008. doi: 10.1371/journal.pone.0225008 31703113 PMC6839852

[45] Ulker M Kenangil G. The relation of circulating levels of leptin with cognition in patients with Alzheimer’s disease. Noro Psikiyatr Ars. (2018) 55:211–4. doi: 10.5152/npa.2017.16978 PMC6138232 30224865

[46] Tiwari S Atluri V Kaushik A Yndart A Nair M. Alzheimer’s disease: pathogenesis, diagnostics, and therapeutics. Int J Nanomed. (2019) 14:5541–54. doi: 10.2147/IJN.S200490 PMC6650620 31410002

[47] Sweeney MD Montagne A Sagare AP Nation DA Schneider LS Chui HC. Vascular dysfunction-The disregarded partner of Alzheimer’s disease. Alzheimers Dement. (2019) 15:158–67. doi: 10.1016/j.jalz.2018.07.222 PMC6338083 30642436

[48] Solis E Jr. Hascup KN Hascup ER. Alzheimer’s disease: the link between amyloid-beta and neurovascular dysfunction. J Alzheimers Dis. (2020) 76:1179–98. doi: 10.3233/JAD-200473 PMC7483596 32597813

[49] Toledo JB Arnold SE Raible K Brettschneider J Xie SX Grossman M. Contribution of cerebrovascular disease in autopsy confirmed neurodegenerative disease cases in the National Alzheimer’s Coordinating Centre. Brain. (2013) 136:2697–706. doi: 10.1093/brain/awt188 PMC3858112 23842566

