# Supplementary material for: Impact of serum leptin and adiponectin levels on brain infarcts in patients with mild cognitive impairment and Alzheimer’s disease: a longitudinal analysis
Source: Front Endocrinol (Lausanne). 2024 Apr 15;15:1389014. doi: 10.3389/fendo.2024.1389014 (PMC11056582; doi:10.3389/fendo.2024.1389014)

Supplementary Material

**List of the ethics committees/institutional review boards that approved the ADNI study**

The Ethics committees/institutional review boards that approved the ADNI study are: Albany Medical Center Committee on Research Involving Human Subjects Institutional Review Board, Boston University Medical Campus and Boston Medical Center Institutional Review Board, Butler Hospital Institutional Review Board, Cleveland Clinic Institutional Review Board, Columbia University Medical Center Institutional Review Board, Duke University Health System Institutional Review Board, Emory Institutional Review Board, Georgetown University Institutional Review Board, Health Sciences Institutional Review Board, Houston Methodist Institutional Review Board, Howard University Office of Regulatory Research Compliance, Icahn School of Medicine at Mount Sinai Program for the Protection of Human Subjects, Indiana University Institutional Review Board, Institutional Review Board of Baylor College of Medicine, Jewish General Hospital Research Ethics Board, Johns Hopkins Medicine Institutional Review Board, Lifespan - Rhode Island Hospital Institutional Review Board, Mayo Clinic Institutional Review Board, Mount Sinai Medical Center Institutional Review Board, Nathan Kline Institute for Psychiatric Research & Rockland Psychiatric Center Institutional Review Board, New York University Langone Medical Center School of Medicine Institutional Review Board, Northwestern University Institutional Review Board, Oregon Health and Science University Institutional Review Board, Partners Human Research Committee Research Ethics, Board Sunnybrook Health Sciences Centre, Roper St. Francis Healthcare Institutional Review Board, Rush University Medical Center Institutional Review Board, St. Joseph's Phoenix Institutional Review Board, Stanford Institutional Review Board, The Ohio State University Institutional Review Board, University Hospitals Cleveland Medical Center Institutional Review Board, University of Alabama Office of the IRB, University of British Columbia Research Ethics Board, University of California Davis Institutional Review Board Administration, University of California Los Angeles Office of the Human Research Protection Program, University of California San Diego Human Research Protections Program, University of California San Francisco Human Research Protection Program, University of Iowa Institutional Review Board, University of Kansas Medical Center Human Subjects Committee, University of Kentucky Medical Institutional Review Board, University of Michigan Medical School Institutional Review Board, University of Pennsylvania Institutional Review Board, University of Pittsburgh Institutional Review Board, University of Rochester Research Subjects Review Board, University of South Florida Institutional Review Board, University of Southern, California Institutional Review Board, UT Southwestern Institution Review Board, VA Long Beach Healthcare System Institutional Review Board, Vanderbilt University Medical Center Institutional Review Board, Wake Forest School of Medicine Institutional Review Board, Washington University School of Medicine Institutional Review Board, Western Institutional Review Board, Western University Health Sciences Research Ethics Board, and Yale University Institutional Review Board.

**Supplemental Table 1. Multivariate regression model for leptin levels in MCI and AD subjects**

|  | **β** | **R^2^c (%)** | **95% CI** | **BIF (%)** | **Functional Form** |
| --- | --- | --- | --- | --- | --- |
| **Age** | **.003*** | 0.3 | .000, .006 | 42.1 | Lin |
| **Gender (F)** | **.496*** | 45.5 | .445, .546 | 100 | NA |
| Definitive diagnosis | -.037 |  | -.095, .021 | 4.0 |  |
| **BMI** | **-4.36*** | 48.5 | -4.79, -3.92 | 100 | Non Lin |
| ADAS-Cog | .001 |  | -.024, .004 | 31.7 |  |
| **MMSE** | **-.013*** | 1.5 | -.023, -.003 | 48.2 | Lin |
| Cerebral Infarcts yes/no | .005 |  | -.056, .067 | 4.5 |  |
| Dyslipidemia | .023 |  | -.023, .069 | 12.5 |  |
| **Hypertension** | **.065*** | 4.2 | .017, .114 | 58.5 | NA |

R^2^=0.58; ***** p<.05

R^2^c = global R^2^ contribution; BIF = Bootstrap Inclusion Frequency based on 1000 bootstrap sample; Lin = Linear; NA = Not applicable

**Supplemental Table 2. Multivariate regression for adiponectin in MCI and AD subjects**

|  | **β** | **R^2^c (%)** | **95% CI** | **BIF (%)** | **Functional Form** |
| --- | --- | --- | --- | --- | --- |
| **Age** | **.003*** | 8.2 | -.001, .006 | 64.8 | Lin |
| **Gender (F)** | **.100*** | 28.5 | .058, .143 | 97.7 | NA |
| Definitive diagnosis | .009 |  | -.053, .072 | 3.8 |  |
| **BMI** | **1.52*** | 55 | 1.06, 1.97 | 100 | Non Lin |
| ADAS-Cog | .001 |  | -.002, .004 | 20.8 |  |
| MMSE | -.008 |  | -.013, .010 | 6.2 |  |
| Cerebral Infarcts yes/no | .003 |  | -.051, .058 | 3.0 |  |
| **Dyslipidaemia** | **-.051*** |  | -.091, -.010 | 51.8 | NA |
| Hypertension | .028 | 8.3 | -.013, .070 | 14.0 |  |

R^2^=0.16; ***** p<.05

R^2^c = global R^2^ contribution; BIF = Bootstrap Inclusion Frequency based on 1000 bootstrap sample; Lin = Linear; NA = Not applicable

**Figure legend**

**Supplemental Figure 1. Leptin and adiponectin levels in males and females in the study population**

Boxplot of mean serum leptin (left) and adiponectin (right) concentrations in HC, MCI and AD groups stratified by gender.


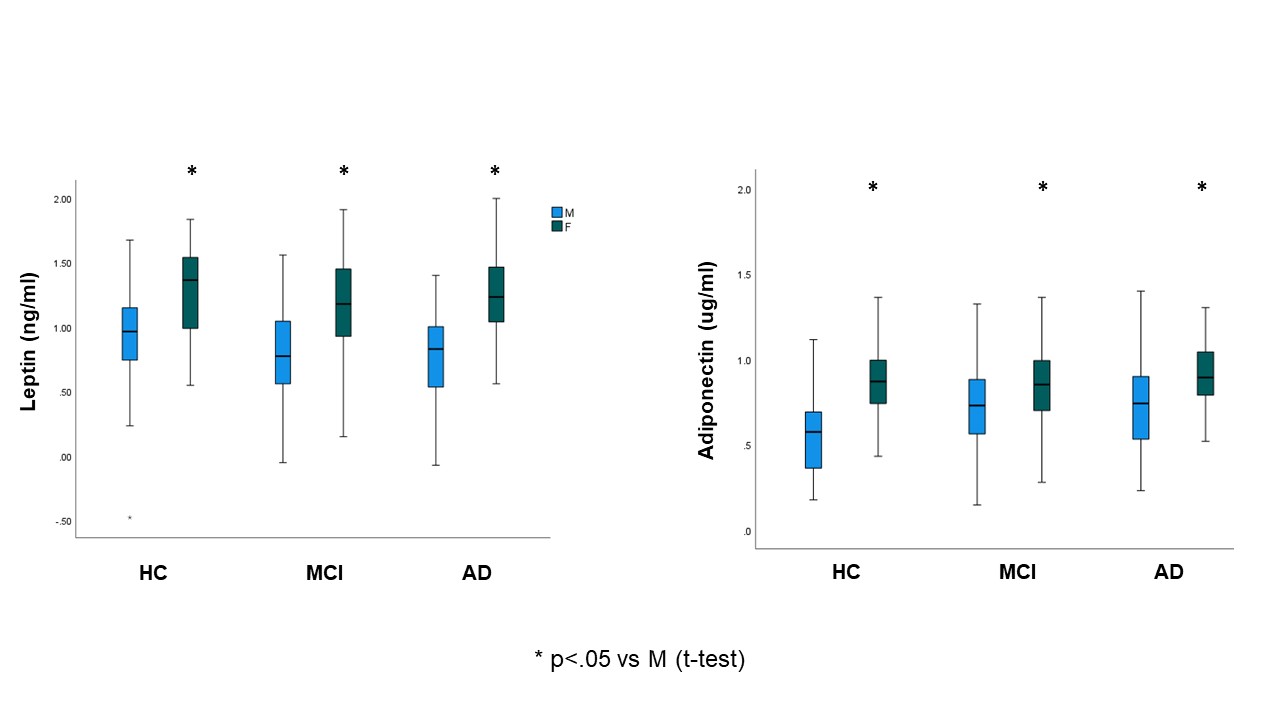

Supplement: Supplementary file 1 [file DataSheet_1.docx]
